# Vanishing Lung Syndrome: An Idiopathic Bullous Emphysema Mimicking Pneumothorax

**DOI:** 10.7759/cureus.9596

**Published:** 2020-08-06

**Authors:** Muhammad N Yousaf, Nim N Chan, Adrien Janvier

**Affiliations:** 1 Internal Medicine, MedStar Union Memorial Hospital, Baltimore, USA; 2 Internal Medicine, MedStar Franklin Square Medical Center, Baltimore, USA; 3 Internal Medicine, MedStar Good Samaritan Hospital, Baltimore, USA; 4 Internal Medicine, MedStar Harbor Hospital, Baltimore, USA; 5 Medicine, MedStar Union Memorial Hospital, Baltimore, USA; 6 Medicine, MedStar Franklin Square Medical Center, Baltimore, USA; 7 Medicine, Medstar Good Samaritan Hospital, Baltimore, USA; 8 Medicine, MedStar Harbor Hospital, Baltimore, USA; 9 Medicine, MedStar Georgetown University, School of Medicine, Washington, DC, USA

**Keywords:** vanishing lung syndrome, giant bulla, emphysema, pneumothorax, copd, idiopathic bullous emphysema, lung volume reduction surgery

## Abstract

Vanishing lung syndrome (VLS) is also referred to as idiopathic giant bullous emphysema and is a rare manifestation of chronic obstructive pulmonary disease (COPD). Middle-aged tobacco smokers, younger marijuana users, and those with alpha-1-antitrypsin deficiency may especially be affected. The clinical and radiographic findings of VLS may initially be misinterpreted as spontaneous pneumothorax. High-resolution CT is the diagnostic imaging modality of choice in these patients and can help to differentiate VLS from pneumothorax. Such imaging also helps guide appropriate management. Management of VLS ranges from a conservative to a surgical approach depending upon patients’ comorbidities and candidacy for surgical resection. We present a case of a 64-year-old man with frequent hospitalizations for COPD exacerbation admitted with worsening shortness of breath and was found to have giant bullae mimicking a pneumothorax on the initial presentation.

## Introduction

Vanishing lung syndrome (VLS), also known as idiopathic giant bullous emphysema represents a rare form of irreversible damage to the pulmonary parenchyma often due to chronic obstructive pulmonary disease (COPD). Patients with this condition typically have a long history of smoking or COPD but may also be younger with a history of marijuana use or have alpha-1 antitrypsin deficiency [1-3]. VLS is associated with a spectrum of clinical manifestations, including worsening dyspnea, cough, declining pulmonary function, and occasionally spontaneous pneumothorax due to ruptured bullae [4]. Robert et al. described the radiological criteria for VLS in 1987 as the presence of giant bullous emphysema predominantly involving one or both upper lung lobes and occupying at least one-third of the hemithorax. These bullae compress the surrounding parenchyma of the lower lung lobes or mediastinum [5]. Subpleural bullae with paraseptal emphysema or centrilobular emphysema may also be seen. Idiopathic giant bullous emphysema may mimic the presentation of pneumothorax with worsening dyspnea, hypoxia, and a chest radiograph revealing an absence of pulmonary markings. However, in the case of a true pneumothorax, a white line representing visceral pleura that has separated from the chest wall is often seen. Clinical concern for pneumothorax should be immediately evaluated with an urgent CT scan of the chest. We present a case of a 64-year-old man with a history of smoking and COPD presenting with clinical and radiographic findings of VLS mimicking a spontaneous pneumothorax on presentation. 

## Case presentation

**Figure 1:**
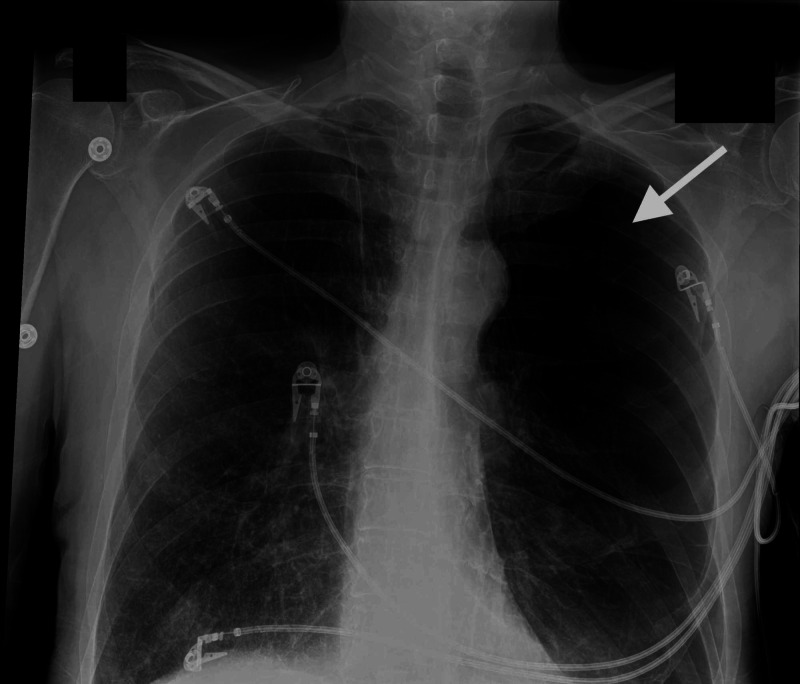
Chest radiograph Chest radiograph (AP view) showing bullous changes involving bilateral upper lobe with severe bullous emphysematous changes particularly in the left hemithorax.

A 64-year-old man with a 50 packs per year smoking history, COPD on three-liter (L) home oxygen, and right lung cancer status post resection 25 years prior to admission presented to the emergency room with worsening shortness of breath, non-productive cough, wheezing, and generalized weakness for the past few weeks. At presentation, vitals were notable for blood pressure of 165/98 mm Hg, tachypnea (RR 22/minute), and oxygen saturation of 92% on 3 L of nasal cannula. On physical examination, he was dyspneic, cachectic appearing with diminished breath sounds more on left hemithorax, and bilateral diffuse wheezing. Laboratory workup was unremarkable. Chest radiograph revealed bullous changes involving both upper lobes and particularly the left hemithorax. Mild blunting of the left costophrenic angle from a small left pleural effusion or pleural thickening was also seen (Figure 1).

**Figure 2:**
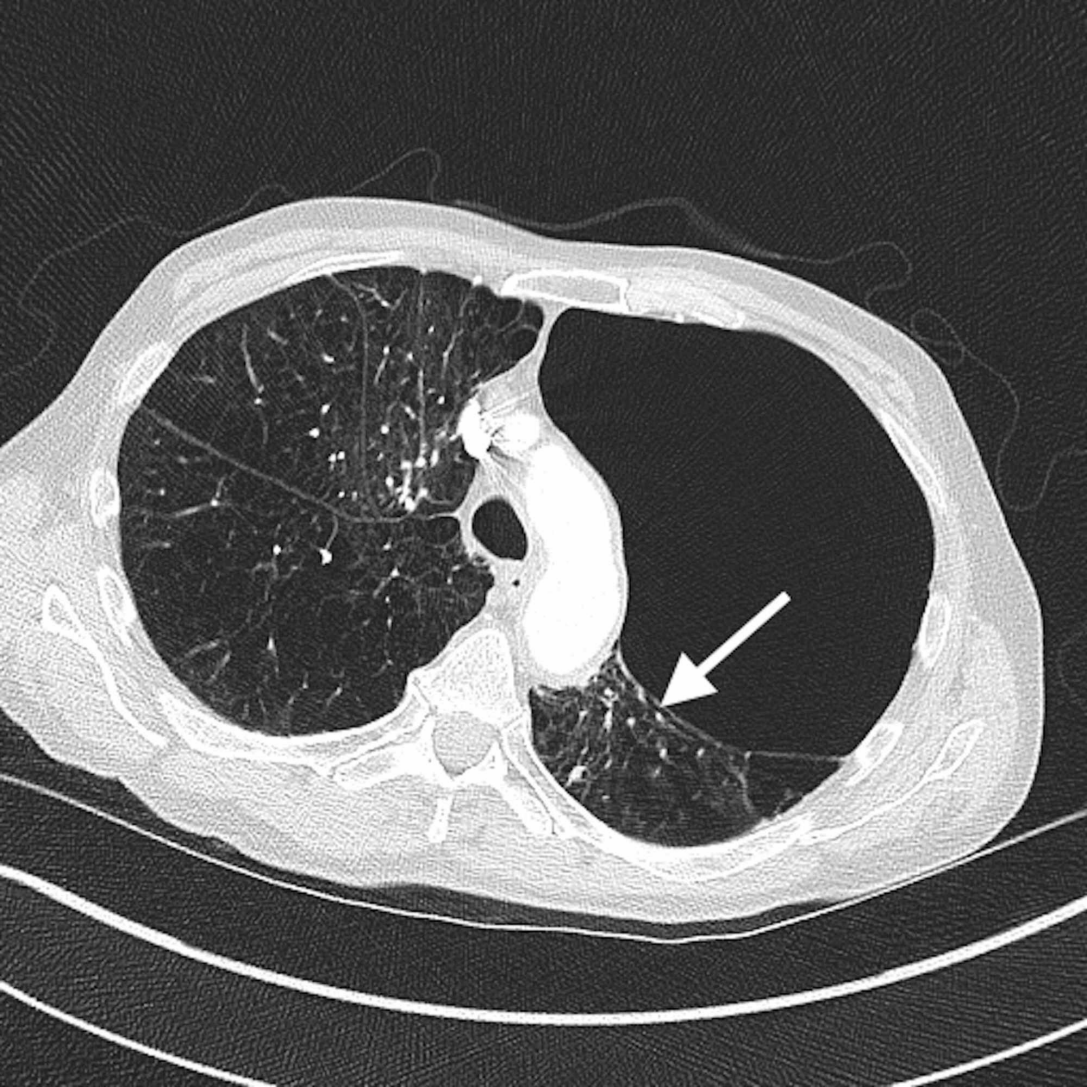
CT scan of the chest Axial section of CT showing severe bilateral emphysematous changes with a giant bulla involving left upper lobe, compressing left lower lobe parenchyma (arrow).

**Figure 3:**
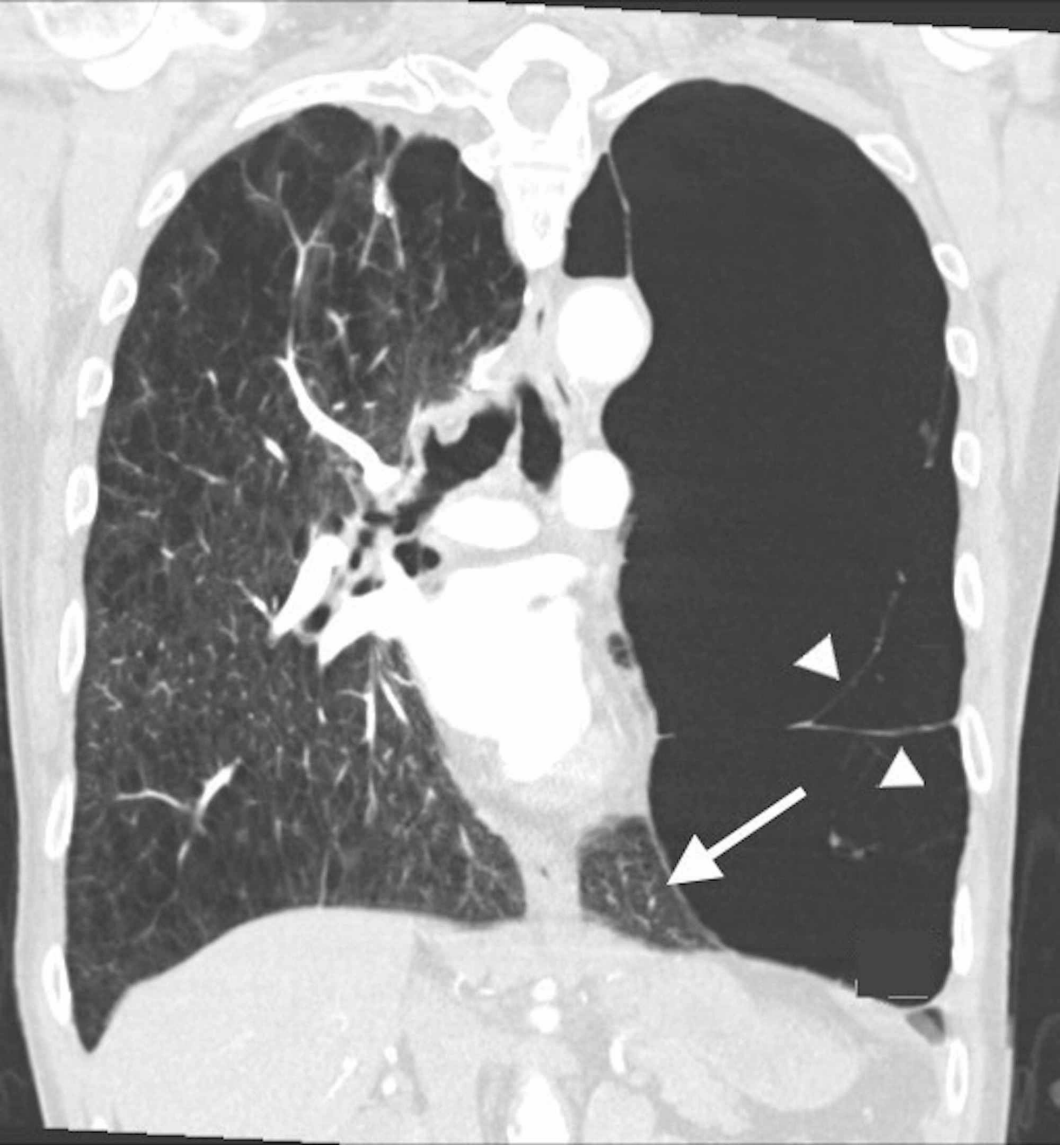
CT scan of the chest Coronal view showing a giant bulla with septae (arrowheads) involving entire left upper lobe of the lung, occupying more than two-third of the left hemithorax resulting in compression of left lower lobe of lung parenchyma (pointed with arrow).

Given his respiratory distress and radiographic findings concerning for a pneumothorax, a CT of the chest was performed and pneumothorax was ruled out. On CT scan, we noted that giant bullae compressed the left lower lung lobe (Figures 2, 3 ). These radiological findings were consistent with VLS that had initially mimicked a pneumothorax. Our patient was not deemed a candidate for lung volume reduction surgery. He improved modestly with oral prednisone and scheduled nebulizer treatments. 

## Discussion

Emphysema is a common manifestation of COPD due to the destruction of pulmonary parenchyma and permanent alveolar dilation. An estimated 11.4% of the world’s population is affected with COPD, and it is the third leading cause of death in the United States [6-8]. Enzymatic destruction of the alveolar wall from elastase produced by alveolar macrophages and neutrophils plays a crucial role in the pathogenesis of bullae formation. Bullae occurring in the setting of emphysema are by definition air-filled cavities greater than 1 cm in diameter. They result in poor gas exchange due to smaller total alveolar surface area and fibrosis of the alveolar membrane itself [9]. These changes lead to a ventilation-perfusion mismatch that results in hypoxemia and hypercapnia. In some cases, enlarging bullae cause compression of the surrounding lung parenchyma and mediastinum. 

For both symptomatic and asymptomatic patients, workup includes pulmonary function testing and a serum alpha-1-antitrypsin level. However, differentiating a pneumothorax from VLS is important because the management differs. In asymptomatic patients with VLS, a more conservative approach employing nebulized bronchodilators is reasonable. However, in symptomatic patients and those with actual pneumothorax, surgical intervention is usually required. The surgical management of VLS includes stapled bullectomy, endocavitary drainage, volume reduction with video-assisted thoracoscopic surgery, one-way endobronchial valves, or lung transplant. In patients undergoing surgical interventions, improvements in pulmonary function (forced expiratory volume in one second [FEV1] and FEV1/forced vital capacity [FVC] ratio), subjective dyspnea scoring, and quality of life resulted [10]. While there is no improvement in the mortality, however, a decreased risk of infection and pneumothorax is associated with this approach [10]. Our patient was not deemed a candidate for the lung volume reduction surgery and was, therefore, managed conservatively. 

## Conclusions

Differentiating VLS from pneumothorax is essential to determining the best plan of care. Acute respiratory distress with hypoxemia may be the early manifestation of pneumothorax due to ruptured giant bullae, and prompt chest tube placement is critical. In VLS, lung volume reduction surgery improves patients’ symptoms, pulmonary function, quality of life, and the need for re-hospitalization. In general, patients should be counseled to stop smoking tobacco and marijuana. Those who are poor candidates for surgical resection may benefit from conservative management or palliation. 
